# Synchronizations in Complex Systems Dynamics Through a Multifractal Procedure

**DOI:** 10.3390/e27060647

**Published:** 2025-06-17

**Authors:** Vlad Ghizdovat, Diana Carmen Mirila, Florin Nedeff, Dragos Ioan Rusu, Oana Rusu, Maricel Agop, Decebal Vasincu

**Affiliations:** 1 Biophysics and Medical Physics Department, “Grigore T. Popa” University of Medicine and Pharmacy, 700115 Iasi, Romania; vlad.ghizdovat@umfiasi.ro; 2 Department of Environmental Engineering, Mechanical Engineering and Agritourism, Faculty of Engineering, “Vasile Alecsandri” University of Bacău, 600115 Bacău, Romania; miriladiana@ub.ro (D.C.M.); florin_nedeff@ub.ro (F.N.); drusu@ub.ro (D.I.R.); 3 Faculty of Material Science and Engineering, “Gheorghe Asachi” Technical University, 700050 Iasi, Romania; oana.rusu@tuiasi.ro; 4 Physics Department, “Gheorghe Asachi” Technical University, Prof. Dr. Docent Dimitrie Mangeron Rd., No. 59A, 700050 Iasi, Romania; 5 Academy of Romanian Scientists, 050044 Bucharest, Romania; 6 Biophysics Department, Faculty of Dental Medicine, “Grigore T. Popa” University of Medicine and Pharmacy, 700115 Iasi, Romania; decebal.vasincu@umfiasi.ro

**Keywords:** multifractal theory of motion, scale relativity theory, complex systems dynamics, Schrödinger multifractal scenario, synchronizations

## Abstract

The dynamics of complex systems often exhibit multifractal properties, where interactions across different scales influence their evolution. In this study, we apply the Multifractal Theory of Motion within the framework of scale relativity theory to explore synchronization phenomena in complex systems. We demonstrate that the motion of such systems can be described by multifractal Schrödinger-type equations, offering a new perspective on the interplay between deterministic and stochastic behaviors. Our analysis reveals that synchronization in complex systems emerges from the balance of multifractal acceleration, convection, and dissipation, leading to structured yet highly adaptive behavior across scales. The results highlight the potential of multifractal analysis in predicting and controlling synchronized dynamics in real-world applications. Several applications are also discussed.

## 1. Introduction

Scale relativity theory, formulated by Laurent Nottale [1], extends the conventional relativity concept by integrating variations in location and velocity alongside alterations in the scale of observation. Nottale hypothesized that, rather than assuming spacetime is smooth and differentiable at all sizes, it may be inherently fractal, particularly at extremely small scales. In a fractal (or multifractal) space, structures exhibit self-similar repetition; however, with variances that result in a range of scaling behaviors instead of a singular fractal dimension.

Scale relativity theory fundamentally asserts that the rules of physics must remain invariant not just under changes in location or time, as proposed in Einstein’s relativity, but also with transformations of scale. Within this paradigm, the conventional smooth trajectories of particles are replaced with fractal, non-differentiable routes. These erratic, self-similar trajectories inherently incorporate a level of unpredictability that reflects the probabilistic characteristics seen in quantum physics. Quantum uncertainty may be an emergent characteristic of a fundamental fractal structure of spacetime [1].

The multifractal dimension enhances the complexity of this depiction. In contrast to a basic fractal, which is defined by a singular scaling exponent, a multifractal space presents a comprehensive range of exponents [2,3]. This indicates that various locations or scales within the same space may exhibit distinct behaviors. This approach is especially attractive for comprehending complex systems [4] because several scaling principles appear to function simultaneously. Within the scale relativity paradigm, this multifractal geometry suggests that the effective laws of physics may vary depending on the scale at which the system is examined. At very small scales, the multifractal characteristics of spacetime may lead to quantum events, yet at larger scales, when spacetime seems more uniform, classical physics prevails.

The scale relativity theory’s potential to provide a geometrical foundation for quantum physics is particularly exciting. Scale relativity ascribes the ostensibly random, probabilistic behavior of particles to the fractal geometry of spacetime [5], offering a deterministic foundation that seems stochastic solely because of the intricate, scale-dependent architecture of space. In a multifractal context, the interaction of many scaling regimes may account for the intricate array of interactions and phenomena observed at numerous scales, from the subatomic level to galactic structures.

Furthermore, the concept of scale invariance aligns with principles in other domains of physics, like the renormalization group in quantum field theory [6]. In such a context, physical constants and interactions appear to “flow” with the scale of observation, suggesting an underlying fractal architecture. Scale relativity theory advances the notion that the fundamental structure of spacetime is multifractal, consequently affecting the behavior of forces and particles at varying scales [1,5,7].

In addition to fundamental physics, the concepts of scale and multifractality have been used across several domains, including astronomy [8], economics [9], and biology [10].

In this context, the Multifractal Theory of Motion provides a novel framework for comprehending the dynamics of systems with movements that exceed the complexity of conventional smooth, deterministic trajectories [11]. This theory asserts that motion is dictated by a multifractal structure, characterized by a hierarchy of scaling behaviors that vary over various segments of the trajectory, rather than supposing a uniform and differentiable route for the item.

The core of the theory posits that the trajectories of moving objects are intrinsically diverse. Unlike classical models that rely on a singular scaling rule or fractal dimension to characterize a system, the multifractal approach acknowledges that several portions of a motion path may display unique scaling characteristics. This range of scaling exponents illustrates a more intricate representation of reality, wherein abnormalities and oscillations across several scales are not mere random noise, but fundamental attributes of the underlying dynamics [11].

A key finding of the Multifractal Theory of Motion is its capacity to reconcile deterministic and stochastic behaviors in dynamical systems. Conventional mechanics frequently regard departures from smooth trajectories as perturbations or noise. Nevertheless, the multifractal viewpoint indicates that these aberrations are intrinsic characteristics of motion. Within this approach, behaviors deemed “chaotic” or “random” are fundamentally supported by a systematic, if intricate, array of scaling principles. This methodology elucidates why some systems, including turbulent flows and certain quantum events, display behavior that resists straightforward prediction; the complexity is not coincidental but fundamentally embedded in the multifractal characteristics of their dynamics [11].

Moreover, the theory emphasizes the significance of scale invariance in nature. Although the scaling parameters may alter throughout various parts of a trajectory, some statistical characteristics stay constant when the system is examined at different magnifications. This invariance is characteristic of fractal and multifractal systems and functions as an effective instrument for linking microscopic dynamics to macroscopic behavior [12,13].

The Multifractal Theory of Motion represents a new approach in the endeavor to comprehend complex systems. In this framework, we apply the Multifractal Theory of Motion to explore synchronization phenomena in complex systems.

## 2. A Short Reminder on the Multifractal Theory of Motion

If any complex system can be assimilated both structurally and functionally to mathematical objects of fractal/multifractal type [1,14,15], any of its dynamics can be described through the Multifractal Theory of Motion [11] by means of continuous and non-differentiable curves (fractal/multifractal curves). Then, the scale covariant derivative [16] becomes functional

(1) $$\frac{\hat{d}}{dt}=\partial t+{\hat{V}}^{e}\partial_{e}+D^{ie}\partial_{i}\partial_{e},\;i,e=1,2,3$$

where the subsequent notations are employed:

(2) $$\partial_{t}=\frac{\partial }{\partial t},\;\partial_{e}=\frac{\partial }{\partial x^{e}},\;\partial_{i}\partial_{e}=\frac{\partial }{\partial x^{i}}\frac{\partial }{\partial x^{e}}\;{\hat{V}}^{e}=V_{D}^{e}-iV_{F}^{e},\;i=\sqrt{-1}\;D^{ie}={\frac{1}{4}\left(dt\right)}^{\left[\frac{2}{f\left(\alpha \right)}\right]-1}\left(d^{ie}+i{\bar{d}}^{ie}\right)\;d^{ie}=\left(\lambda_{+}^{i}\lambda_{+}^{e}-\lambda_{-}^{i}\lambda_{-}^{e}\right),\;{\bar{d}}^{ie}=(\lambda_{+}^{i}\lambda_{+}^{e}+\lambda_{-}^{i}\lambda_{-}^{e})$$

The quantities from Equations (1) and (2) have the following meanings:

$x^{e}$ defines the spatial coordinates, depicted by continuous and nondifferentiable mathematical functions that rely on the scale resolution.

The temporal coordinate, *t*, is depicted by continuous and differentiable mathematical functions that do not rely on scale resolution.

$V_{D}^{e}$ defines the differentiable velocity (i.e., the velocity at differential scale resolution)

$V_{F}^{e}$ illustrates the nondifferentiable velocity (i.e, the velocity at nondifferentiable scale resolution).

$\lambda_{+}^{e}$ and $\lambda_{-}^{e}$ are particular constants of the differentiable-nondifferentiable scale transition coupled with the forward and backward complex systems dynamics, respectively.

The singularity spectrum of order α, identified through $f\left(\alpha \right)$, is defined by $\alpha =\alpha \left(D_{F}\right)$, where $D_{F}$ represents the fractal dimension of the motion curves of the structural units of any complex system [17,18,19].

In this frame of reference, employing the singularity spectrum of order α to characterize complex systems dynamics has obvious advantages:

The dynamics pertaining to the structural units of the complex system may reveal a dominant fractal dimension, which may help in pinpointing a global release pattern which is consistent with a particular global complex system structurality and functionality.

The dynamics pertaining to the structural units of the complex system could include a “collection” of fractal dimensions, which may help in pinpointing local release patterns that are compatible with particular, zone-specific complex structures and functions.

Employing the α-order singularity spectrum, one can pinpoint universality classes in the domain of complex system dynamics, even when the attractors related to said dynamics have distinct aspects.

The explicit configuration of the tensor $D^{ie}$, i.e., the one connected to the fractal-non-fractal/multifractal-non-multifractal scale transitions, is prescribed by fractalization through stochasticity. A very usual procedure of fractalization/multifractalization is by means of stochastic processes [16]. In such a conjecture, it is essential to explain that the stochastic or chaotic processes employed in the study of complex systems dynamics can be split into two categories. The first category comprises homogeneous complex systems dynamics, which are described through a single global fractal dimension and display the identical scaling properties in any time interval (known as fractal processes, such as fractional Brownian type processes). The second category entails complex systems dynamics that can be depicted by employing multiple fractal dimensions and quantifying strictly local singularities, known as multifractal processes.

As such, it is possible to identify the subsequent types of complex systems dynamics:

i. Multifractal complex systems dynamics by means of Markov stochasticity, dictated through the subsequent boundaries [16]:

(3) $$\lambda_{+}^{i}\lambda_{+}^{e}=\lambda_{-}^{i}\lambda_{-}^{e}=-2\lambda \delta^{ie}$$

where λ is a diffusion-type coefficient coupled with the multifractal-non-multifractal scale transition and $\delta^{ie}$ is the Kronecker pseudotensor. Therefore,

(4) $$D^{ie}\to i\lambda {\left(dt\right)}^{\left[\frac{2}{f\left(\alpha \right)}\right]-1}$$

in a way that Equation (1) emerges as below:

(5) $$\frac{\hat{d}}{dt}=\partial_{t}+{\hat{V}}^{e}\partial_{e}-i\lambda {\left(dt\right)}^{\left[\frac{2}{f\left(\alpha \right)}\right]-1}\partial^{e}\partial_{e}$$

ii. Multifractal complex systems dynamics through non-Markov stochasticity dictated by the subsequent boundaries [20]:

(6) $$d^{ie}=4\alpha \delta^{ie},\;\;\;\;\;\;{\bar{d}}^{ie}=-4{\beta \delta }^{ie}$$

where α and β are diffusion-type coefficients coupled with the multifractal-non-multifractal scale transition. As such, the following case results:

(7) $$D^{ie}=\left(\alpha -i\beta \right){\left(dt\right)}^{\left[\frac{2}{f\left(\alpha \right)}\right]-1},$$

in a way that Equation (1) emerges as such:

(8) $$\frac{\hat{d}}{dt}=\partial_{t}+{\hat{V}}^{e}\partial_{e}-(\alpha -i\beta ){\left(dt\right)}^{\left[\frac{2}{f\left(\alpha \right)}\right]-1}\partial^{e}\partial_{e}$$

Now, presuming the functionality of the principle of scale covariance, based on which the laws of physics stay invariant regarding both spatial and temporal transformations and scale transformations, various conservation laws can be built.

In what follows, we restrict our analysis to the implications of operator (5) in the dynamics of complex systems.

## 3. Multifractal Schrödinger-Type Scenario for Analyzing Complex Systems Dynamics

Accepting the functionality of the scale covariance principle (see [1,11]), i.e., applying operator (5) to the complex velocity fields from Equation (2), the motion equation (i.e., the geodesics equation in a multifractal space-time) takes the following form:

(9) $$\frac{\hat{d}{\hat{V}}^{i}}{dt}=\partial_{t}{\hat{V}}^{i}+{\hat{V}}^{e}\partial_{e}{\hat{V}}^{i}-i\lambda {\left(dt\right)}^{\left[\frac{2}{f\left(\alpha \right)}\right]-1}\partial^{e}\partial_{e}{\hat{V}}^{i}=0$$

This means that, for any complex systems dynamics, the multifractal acceleration, $\partial_{t}{\hat{V}}^{i}$, the multifractal convection, ${\hat{V}}^{e}\partial_{e}{\hat{V}}^{i}$, and the multifractal dissipation, $i\lambda {\left(dt\right)}^{\left[\frac{2}{f\left(\alpha \right)}\right]-1}\partial^{e}\partial_{e}{\hat{V}}^{i}$, make their balance at any point on the multifractal curve.

For irrotational motions of the complex systems dynamics, the complex velocity fields from Equation (2) become:

(10) $${\hat{V}}^{i}=-2i\lambda {\left(dt\right)}^{\left[\frac{2}{f\left(\alpha \right)}\right]-1}\partial^{i}ln\psi$$

where ψ is the state function (on the significance of ψ, see [1,11].

In these conditions, by substituting Equation (10) in Equation (9), and using the mathematical procedures from [21], the geodesic Equation (9) takes the form of the multifractal Schrödinger equation:

(11) $${\lambda^{2}\left(dt\right)}^{\left[\frac{4}{f\left(\alpha \right)}-2\right]}\partial^{l}\partial_{l}\psi +i{\lambda \left(dt\right)}^{\left[\frac{2}{f\left(\alpha \right)}-1\right]}\partial_{t}\psi =0$$

Therefore, for the complex velocity field (7), any complex system dynamics can be described through multifractal-type Schrödinger regimens (i.e., Schrödinger-type equations at various scale resolutions).

We note that, for $\lambda =\hbar /2m_{0}$ (where ℏ is the reduced Planck constant and $m_{0}$ is the complex system’s structural unit rest mass) and $D_{F}=2$ [1]; the monofractal dynamics of complex systems can be characterized by Peano-type curves. Then, Equation (8) is reduced to Schrödinger’s differential equation from quantum mechanics [22].

## 4. Synchronized Dynamics in Complex Systems

The differential Equation (11), in the one-dimensional case, remains invariant with respect to the transform group

(12) $$t^{\prime }=\frac{\alpha t+\beta }{\gamma t+\delta },\;x^{\prime }=\frac{x}{\gamma t+\delta },\;\alpha ,\beta ,\gamma ,\delta \in R$$

Now, let us reconsider the first Equation (12), which represents the homographic action of the generic matrix:

(13) $$\hat{M}=\left(\begin{array}{cc} \alpha & \beta \\ \gamma & \delta \end{array}\right)$$

In this context, let us focus on the following: a relation must be established between the ensemble of matrices $\hat{M}$ and the ensemble of values corresponding to t, for which $t^{\prime }$ remains constant.

From a geometric standpoint, this entails identifying the collection of points $\left(\alpha ,\beta ,\gamma ,\delta \right)$, univocally corresponding to the values of the parameter t. By using the first Equation (12), along with the methods from [16,21] (i.e., Riccati-type gauge), we obtain

(14) $$dt+\omega_{1}t^{2}+\omega_{2}t+\omega_{3}=0$$

where the following notations are used [11,23]:

(15) $$\omega_{1}=\frac{\gamma d\alpha -\alpha d\gamma }{\alpha \delta -\beta \gamma },\;\omega_{2}=\frac{\delta d\alpha -\alpha d\delta +\gamma d\beta -\beta d\gamma }{\alpha \delta -\beta \gamma },\;\omega_{3}=\frac{\delta d\beta -\beta d\delta }{\alpha \delta -\beta \gamma }$$

It is readily apparent that the metric

(16) $$ds^{2}=\frac{{\left(\alpha d\delta +\delta d\alpha -\beta d\gamma -\gamma d\beta \right)}^{2}}{4{\left(\alpha \delta -\beta \gamma \right)}^{2}}-\frac{d\alpha d\delta -d\beta d\gamma }{\alpha \delta -\beta \gamma }$$

is in a direct relation with the discriminant of the quadratic polynomial from Equation (14)

(17) $$ds^{2}=\frac{1}{4}\left({\omega_{2}}^{2}-4\omega_{1}\omega_{2}\right)$$

We must point out the fact that, for a particular case of 1-forms $\omega_{1},\omega_{2},\omega_{3}$ [11], the metric (17) also admits the SL(2R) invariance.

The three differential forms from Equation (15) can provide a coframe [11] in any point of the absolute space. This facilitates the conversion of the geometric characteristics of absolute space into algebraic qualities associated with differential Equation (14).

In this case, the 1-forms $\omega_{1},\omega_{2},\omega_{3}$ are differentiated exactly in the same parameter τ of the geodesic. Along these geodesics, Equation (14) is transformed into a Riccati-type differential equation (Riccati-type gauge):

(18) $$\frac{dt}{d\tau }=P\left(t\right),\;P\left(t\right)=c_{1}t^{2}+2c_{2}t+c_{3}$$

We will now discuss the in-phase correlative dynamics of complex systems.

Now, the parameters $c_{1}$, $c_{2}$, $c_{3}$ are constants which characterize a certain geodesic of the family. In this case, by employing the method from [11,16], Equation (18) highlights dynamics correlated by a Stoler transform [24]:

(19) $$z=-\frac{c_{2}}{c_{1}}+\frac{\omega }{c_{1}}\left[\frac{2r\sin\left[2\omega \left(\tau -\tau_{0}\right)\right]}{1+r^{2}+2r\cos\left[2\omega \left(\tau -\tau_{0}\right)\right]}+i\frac{1-r^{2}}{1+r^{2}+2r\cos\left[2\omega \left(\tau -\tau_{0}\right)\right]}\right]$$

where r and $\tau_{0}$ are two real constants, specific to the solution.

**Figure 1:**
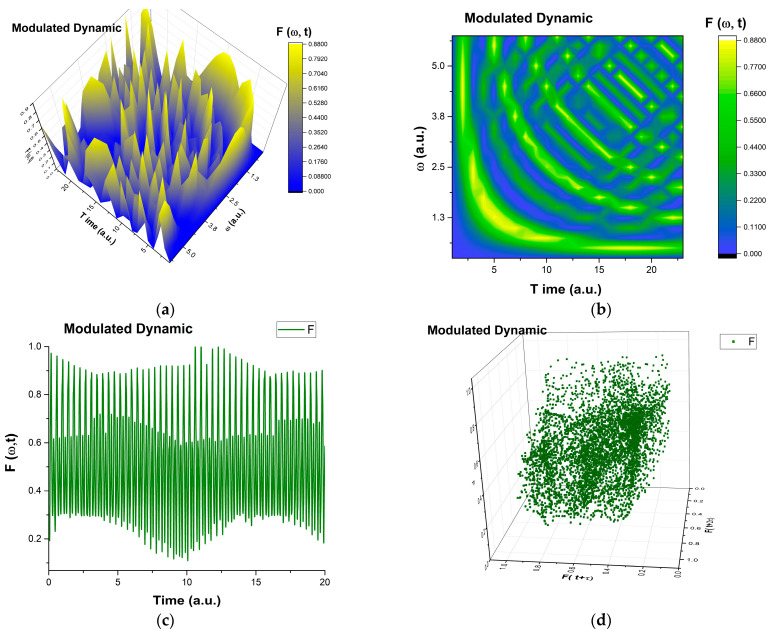
(**a**–**d**) 3D plot (**a**), contour plot (**b**), time series (**c**), and reconstructed attractor (**d**) for solution $Re\left(zc_{1}+c_{2};c_{1}=c_{2}\equiv 1\right)\equiv F\left(\omega ,\tau =time\right)$ corresponding to the maximum value of the pulsation-type characteristic $\omega_{max}=6$.

We present, in Figure 1, the explication of modulated dynamics in complex systems, based on Equation (19), through $F\left(\omega ,\tau =time\right)$, for $\omega_{max}=6$, r=1 and $\tau_{0}=0$.

## 5. Discussions

In the following, we want to mention several domains in which our theoretical model can be applied.

In medicine, feedback-control principles are used to regulate physiological dynamics—for instance, pacemakers modulate cardiac rhythms, and insulin pumps use closed-loop control to stabilize blood glucose. In neuroscience, dynamical models (e.g., Wilson–Cowan equations) help explain how networks of excitatory and inhibitory neurons produce oscillatory brain activity and cognitive functions [25]. This insight guides treatments like deep brain stimulation, which aims to modulate abnormal neural dynamics (such as Parkinsonian tremors) back to healthy patterns.

In ecology, population dynamics models (like the Lotka–Volterra predator–prey equations) demonstrate how species interactions lead to cyclical oscillations in population sizes [26,27]. Such models inform wildlife management and conservation—for example, predicting how predator introduction or resource changes will modulate population cycles over time. By combining nonlinear dynamics theory with empirical data, researchers can design interventions (drugs, stimuli, or policies) to steer biological systems toward desired states or mitigate undesirable oscillations (such as epidemics)

On cosmic scales, modulated dynamics describe how astronomical structures and phenomena evolve under gravitational interactions. Theoretical frameworks like general relativity and cosmological perturbation theory are essential for modeling these dynamics. Cosmological perturbation theory, for example, explains how tiny density fluctuations in the early universe grew under gravity to form larger structures—ultimately seeding the formation of stars, galaxies, and clusters [28]. Since gravity is nonlinear, small initial perturbations become amplified, and nonlinear dynamics take over as structures collapse and merge. General relativity further predicts dynamic phenomena such as gravitational waves [29]. To tackle strongly nonlinear cosmic events (e.g., galaxy collisions or black hole mergers), astrophysicists rely on numerical simulations and relativistic models, since analytic perturbation methods break down in these regimes [30].

Atmospheric systems exhibit richly modulated dynamics across a wide range of scales, from microscopic turbulent eddies to planetary-scale circulation patterns. The foundation is classical fluid dynamics: the atmosphere’s behavior is governed by nonlinear Navier–Stokes equations [31] (with effects of Earth’s rotation and heating), which yield phenomena like jet streams, cyclones, and ocean-atmosphere oscillations. A hallmark of atmospheric dynamics is its chaotic nature. Chaos theory was, in fact, born from atmospheric modeling when Edward Lorenz discovered that tiny changes in initial conditions can lead to vastly different weather outcomes—the famous butterfly effect [32]. This sensitive dependence on initial state means atmospheric flows are inherently unpredictable beyond a certain time horizon, yet they still exhibit structured oscillatory modes. For instance, large-scale waves in the atmosphere (e.g., Rossby waves and jet stream meanders) can propagate and interact, transferring energy across regions and modulating weather patterns [33]. Turbulence is another key aspect: turbulent cascades distribute energy from large scales (solar-heated currents) to small scales, creating ever-changing, fractal-like motion. As a consequence, atmospheric dynamics are being analyzed by a mix of fluid mechanics, nonlinear wave interactions, and chaotic theory, which together explain both the regular cyclic phenomena (like seasonal cycles and oscillations) and the irregular, rapidly changing behavior of weather. Therefore, understanding atmospheric modulated dynamics is crucial for practical applications in weather and climate science. Weather prediction relies on numerical models that integrate fluid dynamics equations forward in time; understanding chaos has led to using ensemble forecasts (running models multiple times with slight variations) to estimate the range of possible outcomes [34,35].

Taking the above into account, our multifractal framework can be experimentally tested across various domains, specifically:

Biological Systems: Future research could involve experimental validation through high-resolution physiological measurements. Techniques such as electrocardiography (ECG) to analyze heart rate variability or electroencephalography (EEG) to monitor neural activity can test multifractal synchronization predictions. Applying methods such as wavelet transform modulus maxima (WTMM) or detrended fluctuation analysis (DFA) could quantify multifractality experimentally [36,37].

Ecological Systems: Controlled predator-prey experiments could directly observe multifractal oscillatory behaviors predicted by our theory. Using population dynamics models (e.g., Lotka–Volterra systems), combined with precise monitoring technologies like automated imaging and telemetry tagging, can provide data to analyze multifractal patterns in population dynamics [38,39].

Atmospheric Systems: Advanced satellite and ground-based observational data, including radar measurements and Doppler lidar techniques, can empirically validate the multifractal nature of atmospheric turbulence and weather patterns. Satellite imagery analysis utilizing multifractal detrended fluctuation analysis (MFDFA) and spatial correlation methods can substantiate predictions on scale-dependent atmospheric dynamics [40,41].

Moreover, our framework can be enriched by explicitly considering the multifractal singularity spectrum through the lens of generalized entropies such as Rényi entropy, Shannon entropy [42,43], and related information theory metrics.

Multifractal singularity spectra, as discussed in our manuscript, quantify the distribution and intensity of scaling behaviors across different scales. This distribution inherently represents how information is stored, transferred, or dissipated within complex systems. In our opinion, a further development of our model could provide insights into how multifractal scaling properties correlate with information complexity, predictability, and uncertainty within complex systems. From such a perspective, our future research will focus on the following related topics:

- Detailed discussions linking multifractal measures to generalized Rényi entropy and its interpretation regarding uncertainty and information storage across scales;
- Applications of Shannon’s entropy and mutual information to determine how synchronization dynamics relate to informational exchanges within complex systems;
- Analyzing divergence measures, such as the Kullback–Leibler divergence [44], to quantify differences between multifractal distributions and their theoretical predictions, reflecting informational discrepancies or system dynamics deviations.

## 6. Conclusions

This paper presents a way for successfully analyzing synchronization in the dynamics of complex systems using a multifractal approach. By expanding the tenets of scale relativity theory and the Multifractal Theory of Motion, we have shown that complex systems display behavior fundamentally influenced by the interaction between deterministic rules and stochastic multifractal structures.

Our findings highlight that complex systems do not conform to a singular fractal dimension but rather display a range of scaling characteristics. The multifractal nature enables a more nuanced characterization of complex system dynamics, wherein fluctuations and changes across several scales contribute to emergent collective behaviors. We have demonstrated that the dynamics of complex systems may be articulated using multifractal Schrödinger-type equations, elucidating an inherent relationship between quantum mechanics and the fractal geometry of spacetime. Additionally, by elucidating synchronization processes within these systems, we establish a theoretical framework for comprehending the interaction and self-organization of diverse scales of motion into coherent structures.

Future research should concentrate on enhancing analytical tools for multifractal analysis, advancing computer models for deriving multifractal spectra, and investigating the relationships between scale invariance and basic physical laws. Furthermore, experimental confirmation of the suggested multifractal synchronization mechanisms will be essential for connecting theoretical insights with empirical results.

The multifractal viewpoint on complex system dynamics offers a robust foundation for comprehending the sophisticated and self-organized behaviors seen in several scientific fields. By adopting the ideas of scale covariance and synchronization, we get a more cohesive and predictive comprehension of the evolution, interaction, and adaptation of complex systems across many scales.

## Data Availability

All the data are presented in the manuscript.
